# Nutrient Status and Supplement Use During Pregnancy Following Metabolic Bariatric Surgery: A Multicenter Observational Cohort Study

**DOI:** 10.1007/s11695-024-07446-4

**Published:** 2024-08-14

**Authors:** Laura Heusschen, Agnes A. M. Berendsen, Arianne C. van Bon, Judith O. E. H. van Laar, Ineke Krabbendam, Eric J. Hazebroek

**Affiliations:** 1 Division of Human Nutrition and Health, Wageningen University, PO Box 17 6700 AA, Wageningen, The Netherlands; 2 Department of Bariatric Surgery, Vitalys, Part of Rijnstate Hospital, PO Box 9555, 6800 TA Arnhem, The Netherlands; 3 Department of Internal Medicine, Rijnstate Hospital, PO Box 9555, 6800 TA Arnhem, The Netherlands; 4 Department of Obstetrics and Gynecology, Máxima Medical Center, PO Box 7777, 5500 MB Veldhoven, The Netherlands; 5 Department of Obstetrics and Gynecology, Hospital Gelderse Vallei, PO Box 9025, 6710 HN Ede, The Netherlands

**Keywords:** Deficiencies, Metabolic Bariatric Surgery, Nutrient status, Pregnancy, Supplementation

## Abstract

**Introduction:**

Pregnant women with a history of metabolic bariatric surgery (MBS) are at high risk of developing nutrient deficiencies, leading to greater challenges to reach nutritional requirements. This study compared nutrient status of women using specialized “weight loss surgery” multivitamin supplementation (WLS-MVS) to those using standard supplementation (sMVS) during pregnancy following MBS.

**Methods:**

Multicenter observational cohort study including 119 pregnant women at 41.0 (18.5–70.0) months after Roux-en-Y gastric bypass (RYGB, *n* = 80) or sleeve gastrectomy (SG, *n* = 39). Routine blood samples were analyzed every trimester (T1, T2, T3), and micronutrient serum levels were compared between WLS-MVS and sMVS users.

**Results:**

During pregnancy after RYGB, WLS-MVS users demonstrated higher serum concentrations of hemoglobin (7.4 [7.2, 7.5] vs. 7.0 [6.8, 7.3] mmol/L), ferritin (23.2 [15.0, 35.7] vs. 13.7 [8.4, 22.4] µg/L), and folic acid (31.4 [28.7, 34.2] vs. 25.4 [21.3, 29.4] nmol/L) and lower serum vitamin B6 levels (T1: 90.6 [82.0, 99.8] vs. 132.1 [114.6, 152.4] nmol/L) compared to sMVS users. Iron deficiencies and elevated serum vitamin B6 levels were less prevalent in the WLS-MVS group. During pregnancy after SG, WLS-MVS users showed higher serum vitamin D concentrations (89.7 [77.6, 101.8] vs. 65.4 [53.3, 77.4] nmol/L) and lower serum vitamin B1 concentrations (T2: 137.4 [124.2, 150.6] vs. 161.6 [149.0, 174.1] nmol/L, T3: 133.9 [120.1, 147.7] vs. 154.7 [141.9, 167.5] nmol/L) compared to sMVS users.

**Conclusion:**

Low maternal concentrations of micronutrients are highly prevalent during pregnancy after MBS. The use of specialized multivitamin supplementation generally resulted in higher serum levels during pregnancy compared to standard supplementation. Future research is needed to investigate how supplementation strategies can be optimized for this high-risk population.

**Graphical Abstract:**

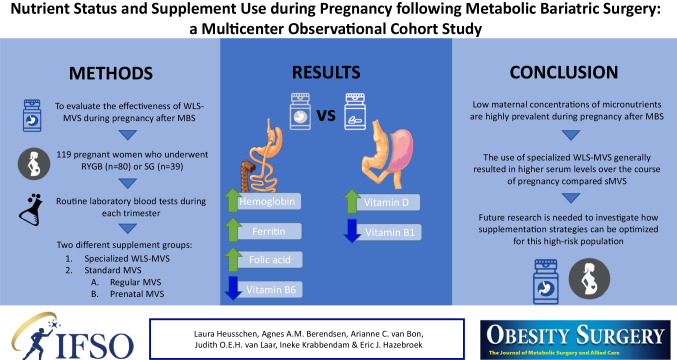

**Supplementary Information:**

The online version contains supplementary material available at 10.1007/s11695-024-07446-4.

## Introduction

Metabolic bariatric surgery (MBS) is the most effective treatment for people with severe obesity, resulting in substantial and long-term weight loss and reduction of obesity-related health risks [1, 2]. More than half of all MBS procedures are performed in women of reproductive age [3], and the Roux-en-Y gastric bypass (RYGB) and sleeve gastrectomy (SG) are the most commonly performed procedures [3]. Undergoing MBS prior to pregnancy significantly reduces the risk of obesity-related complications such as subfertility, gestational diabetes, and hypertensive disorders in pregnancy [4, 5]. However, decreased intake and absorption of nutrients after surgery in combination with the increased demand for nutrients during pregnancy may lead to more pronounced deficiencies [6]. Furthermore, pregnancy symptoms such as morning sickness or hyperemesis gravidarum and abdominal complaints may worsen nutrient status during pregnancy [6, 7]. Overall, low maternal concentrations of vitamins A, B_12_, and D, folic acid, iron, and zinc are frequently reported during pregnancy after MBS [8–10]. Potential neonatal adverse effects that are associated with maternal deficiencies during pregnancy include preterm birth, fetal growth restriction, congenital malformations, and neurological and developmental impairment [6, 7, 9, 11].

Consensus recommendations for prenatal care of these patients have been proposed [12], but evidence-based guidelines regarding optimal nutritional monitoring and supplementation strategies during pregnancy after MBS are lacking. Regular “over-the-counter” or prenatal multivitamin supplements are likely not sufficient to cover the needs of pregnant women who have undergone MBS. Fortunately, specialized “weight loss surgery” multivitamin supplements (WLS-MVS) that are specifically developed for patients after MBS are emerging. The composition of these supplements is often tailored to the type of procedure and varies between brands, but they generally contain high doses of folic acid, vitamins B_12_ and D, elementary iron, and zinc. Although the superiority of these supplements compared to standard multivitamin supplementation (sMVS) has been demonstrated in the general population after MBS [13–15], their efficacy during pregnancy is largely unknown.

Therefore, the aim of this observational cohort study was to explore differences in nutrient status among women using WLS-MVS versus sMVS during pregnancy following MBS.

## Methods

### Study Design and Participants

The NEWBIE study (**N**utritional status of pr**E**gnant **W**omen following **B**ariatr**I**c surg**E**ry) is a multicenter observational cohort study that was conducted from November 2018 until October 2022 at three general hospitals in the Netherlands (Rijnstate Hospital, Arnhem (RHA), Máxima Medical Center, Veldhoven (MMC), Hospital Gelderse Vallei, Ede (HGV)). Within these hospitals, women with a history of MBS are recommended to postpone pregnancy during the period of rapid weight loss (at least 12 months) and to use specialized WLS-MVS. Antenatal care follows a specific protocol recommending supplementation with WLS-MVS and close monitoring of maternal nutrient status as well as fetal growth and complications (e.g., internal herniation).

All pregnant women older than 18 years with a medical history of MBS presenting at the obesity or antenatal clinic were eligible for recruitment. Exclusion criteria were elective termination of pregnancy, multiple pregnancy, MBS procedures other than RYGB or SG, reversal of the MBS procedure, and malnutrition due to other causes (e.g., malignancy, alcoholism). Participants were preferably included before 12 weeks of pregnancy and followed up until 2 months post-partum. A total of 129 participants were included of which three women were excluded because of twin pregnancies (*n* = 2) and history of another MBS procedure (*n* = 1). During data analysis, seven participants were excluded because of insufficient data about pregnancy (*n* = 1), unknown MVS use (*n* = 4), and not using MVS during pregnancy (*n* = 2). The final population for data analysis consisted of 119 participants of whom 80 women after RYGB (67%) and 39 after SG (33%) (Fig. S1).

This study was conducted according to the guidelines laid down in the Declaration of Helsinki and all procedures involving research study participants were reviewed and approved by the institutional ethics committees of the participating hospitals (ref 2018–1267). Written informed consent was obtained from all subjects.

### Data Collection

#### Clinical Parameters

Maternal characteristics (age, geographic origin, educational level, smoking status, anthropometrics, type of MBS, presence of preexisting diabetes or hypertension) and antepartum variables (time to conception, mode of conception, parity, gestational weight gain, pregnancy complications) were collected from the medical records. Educational level was defined as low (primary education and prevocational secondary education), medium (senior general secondary education, preuniversity education, and secondary vocational education), or high (higher vocational education and university). Smoking status was defined as never, former (stopped *before* pregnancy), or current (smoked during pregnancy). Anthropometric measurements including height (m) and body weight (kg) were performed during standard visits. Percent total body weight loss (%TWL) at conception was calculated as body weight loss divided by body weight before surgery, multiplied by 100%.

Time from surgery to conception was defined as the period in months between surgery and conception. Mode of conception was classified as spontaneous or assisted (by use of fertility treatment). Gestational weight gain in kilograms was calculated as the difference between late pregnancy weight (weight at the day of delivery or within less than 4 weeks before delivery) and prepregnancy weight (weight at the first antenatal visit or self-reported weight before pregnancy). Subsequently, gestational weight gain was classified as inadequate, adequate, or excessive based on prepregnancy body mass index (BMI) according to the National Academy of Medicine (NAM) recommendations [16]. Evaluated complications during pregnancy included gestational diabetes mellitus (new-onset diabetes diagnosed by glucose monitoring), hypertensive disorders (new-onset hypertension, above 140/90 mm Hg), hyperemesis gravidarum (severe, persistent nausea and vomiting), and internal herniation (small bowel obstruction).

#### Supplementation Use

All women were advised to use MVS daily, preferably a specialized WLS-MVS that is specifically developed for patients after MBS. Self-reported information on the use of MVS (type, composition, dosage, and compliance) was obtained during each trimester, and participants were categorized as either users of WLS-MVS or sMVS accordingly. sMVS were defined as regular, over-the-counter MVS or prenatal supplements. The composition of the MVS that were most frequently used can be found in Table S1. Participants using both WLS-MVS and sMVS on a daily basis were assigned to the WLS-MVS group, whereas participants who used WLS-MVS and sMVS on alternate days were assigned to the sMVS group. Non-users of MVS were excluded from the analyses.

In addition to daily MVS, all participants were advised to use calcium/vitamin D_3_ supplementation as part of the standard protocol after MBS. According to general recommendations of the Dutch Health Council [17], supplementation of 400 µg folic acid was also recommended in the preconception period until 8 weeks after conception. In case of observed low micronutrient serum levels during pregnancy, a prescription for the required supplementation was provided according to local protocol.

#### Laboratory Evaluation

Standard routine laboratory blood tests were performed during each trimester (T1: week 1–12, T2: week 13–26, T3: week 27–42). Evaluated laboratory parameters slightly differed between the centers, but generally included hemoglobin, ferritin, folic acid, vitamins A, B_1_, B_6_, B_12_, and D, and calcium. Calcium levels were corrected for albumin using the following equation: Ca_corr_ = total calcium + 0.02*(40-albumin). A low serum level was defined as a serum level below the local reference value at the time of blood collection (Table S2) as there were no validated standards available for the required levels of micronutrients during pregnancy, except for hemoglobin [18]. Serum ferritin levels below the reference value were used as a marker for iron deficiency.

### Statistical Analyses

General characteristics are reported as mean ± standard deviation (normal distribution) or as median (Q1–Q3, non-normal distribution) for continuous variables and as frequency (percentage) for categorical variables.

Differences in serum concentrations across the three trimesters of pregnancy between WLS-MVS users and sMVS users were analyzed using linear mixed-effects models. Serum concentrations of ferritin and vitamin B6 were log-transformed before analysis. The crude model consisted of fixed effects for MVS (WLS-MVS, sMVS), trimester (T1, T2, T3), and their interaction term, plus a random effect for participants. Trimester entered the model as a repeated measure using a first-order autoregressive structure. Log-likelihood ratio tests were performed to explore potential confounders including center, smoking status, surgery-to-conception interval, BMI at conception, timing of sampling, and the use of additional supplementation for iron, folic acid, vitamin B_12_, and vitamin D during pregnancy. Final models for RYGB included BMI at conception, use of additional supplementation for ferritin and vitamin B_12_ (yes/no/missing), use of calcium/vitamin D_3_ supplementation for calcium and vitamin D (yes/no/missing), and timing of sampling for vitamin D (in months). Final models for SG included the use of additional supplementation for ferritin (yes/no/missing) and timing of sampling for vitamin D (in months). Serum concentrations measured after intravenous iron infusions (ferritin) and hydroxocobalamin injections (vitamin B_12_) were removed from the analyses to prevent biased estimates. Results are presented as estimated (geometric) marginal mean and 95% confidence intervals (CI). Descriptives of the original serum data at each trimester can be found in Table S3.

The prevalence of low and elevated serum levels at each trimester was analyzed using chi-square tests or Fisher’s exact test (if  more than 20% of expected counts were less than 5) and presented as frequency (percentage).

All statistical analyses were performed separately for the RYGB and SG group, using IBM SPSS Statistics 25 for Windows (IBM Corp., Armonk USA). A two-sided *p* value below 0.05 was considered statistically significant.

## Results

### General Characteristics

Mean age at conception of the total study population was 31.3 ± 4.7 years; the majority of the participants was of West-European origin (92.4%), had a medium educational level (37.8%), and never smoked (61.3%) (Table 1). Median time from surgery to conception was 50.0 (23.4–77.0) months in the RYGB group and 32.2 (16.4–43.8) months in the SG group, and the majority of the participants became pregnant more than 24 months after MBS (RYGB: 75.0%, SG: 59.0%). Mean TWL from surgery to conception was 32.0 ± 9.1% after RYGB and 32.5 ± 8.5% after SG.

**Table 1:** General characteristics of the study population according to type of MBS

Characteristic	Study population (*n* = 119)	RYGB (*n* = 80)	SG (*n* = 39)
Maternal age at conception (years)	31.3 ± 4.7	32.1 ± 4.5	29.7 ± 4.9
Geographic origin (West European)	110 (92.4)	76 (95.0)	34 (87.2)
Highest level of education^a^			
Low	21 (17.6)	16 (20.0)	5 (12.8)
Medium	45 (37.8)	30 (37.5)	15 (38.5)
High	24 (20.2)	15 (18.8)	9 (23.1)
*Missing*	29 (24.4)	19 (23.8)	10 (25.6)
Smoking status			
Never	73 (61.3)	45 (56.3)	28 (71.8)
Former	22 (18.5)	18 (22.5)	4 (10.3)
Current	24 (20.2)	17 (21.3)	7 (17.9)
Preexistent diabetes mellitus	1 (0.8)	0 (0.0)	1 (2.6)
Preexistent hypertension	1 (0.8)	1 (1.3)	0 (0.0)
BMI at conception (kg/m^2^)	28.7 (26.0–32.5)	29.0 (25.9–32.0)	27.7 (26.0–33.0)
TWL from surgery to conception (%)^b^	32.2 ± 8.9	32.0 ± 9.1	32.5 ± 8.5
Time from surgery to conception (months)	41.0 (18.5–70.0)	50.0 (23.4–77.0)	32.2 (16.4–43.8)
< 12 months	11 (9.2)	6 (7.5)	5 (12.8)
12–24 months	25 (21.0)	14 (17.5)	11 (28.2)
> 24 months	83 (69.7)	60 (75.0)	23 (59.0)
Primiparity	57 (47.9)	34 (42.5)	23 (59.0)
Fertility treatment	14 (11.8)	11 (13.8)	3 (7.7)
Gestational weight gain (kg)^c^	10.6 ± 7.2	9.9 ± 6.9	11.9 ± 7.7
Inadequate weight gain	22 (18.5)	16 (20.0)	6 (15.4)
Adequate weight gain	22 (18.5)	15 (18.8)	7 (17.9)
Excessive weight gain	38 (31.9)	25 (31.3)	13 (33.3)
*Missing*	37 (31.1)	24 (30.0)	13 (33.3)
Pregnancy complications			
Gestational diabetes mellitus	6 (5.0)	6 (7.5)	0 (0.0)
Hypertensive disorders	7 (5.9	3 (3.8)	4 (10.3)
Hyperemesis gravidarum	3 (2.5)	0 (0.0)	3 (7.7)
Internal herniation	3 (2.5)	3 (3.8)	–
Use of additional supplementation			
Folic acid	80 (67.2)	55 (68.8)	25 (64.1)
Iron	51 (42.9)	36 (45.0)	15 (38.5)
Vitamin B_12_	37 (31.1)	28 (35.0)	9 (23.1)
Calcium/vitamin D	92 (77.3)	64 (80.0)	28 (71.8)

Data are presented as mean ± SD, median (Q1–Q3) or frequency (percentage)

*RYGB*, Roux-en-Y gastric bypass; *SG*, sleeve gastrectomy; *BMI*, body mass index; *TWL*, total body weight loss

^a^Low education = primary and prevocational secondary education; medium education = senior general secondary education, preuniversity education and secondary vocational education; high education = higher vocational education and university

^b^Missing for *n* = 5 (RYGB)

^c^According to NAM recommendations[16]

### Nutrient Status and Supplement use after RYGB

Throughout pregnancy following RYGB, low maternal serum concentrations were frequently observed for hemoglobin (28.7%), ferritin (60.0%), vitamin B_12_ (43.8%), vitamin A (21.3%), and vitamin D (45.0%) and to a lesser extent for folic acid (12.5%) and calcium (13.8%). Low serum levels of vitamin B_1_ and B_6_ were rare (2.5%).

**Fig. 1:**
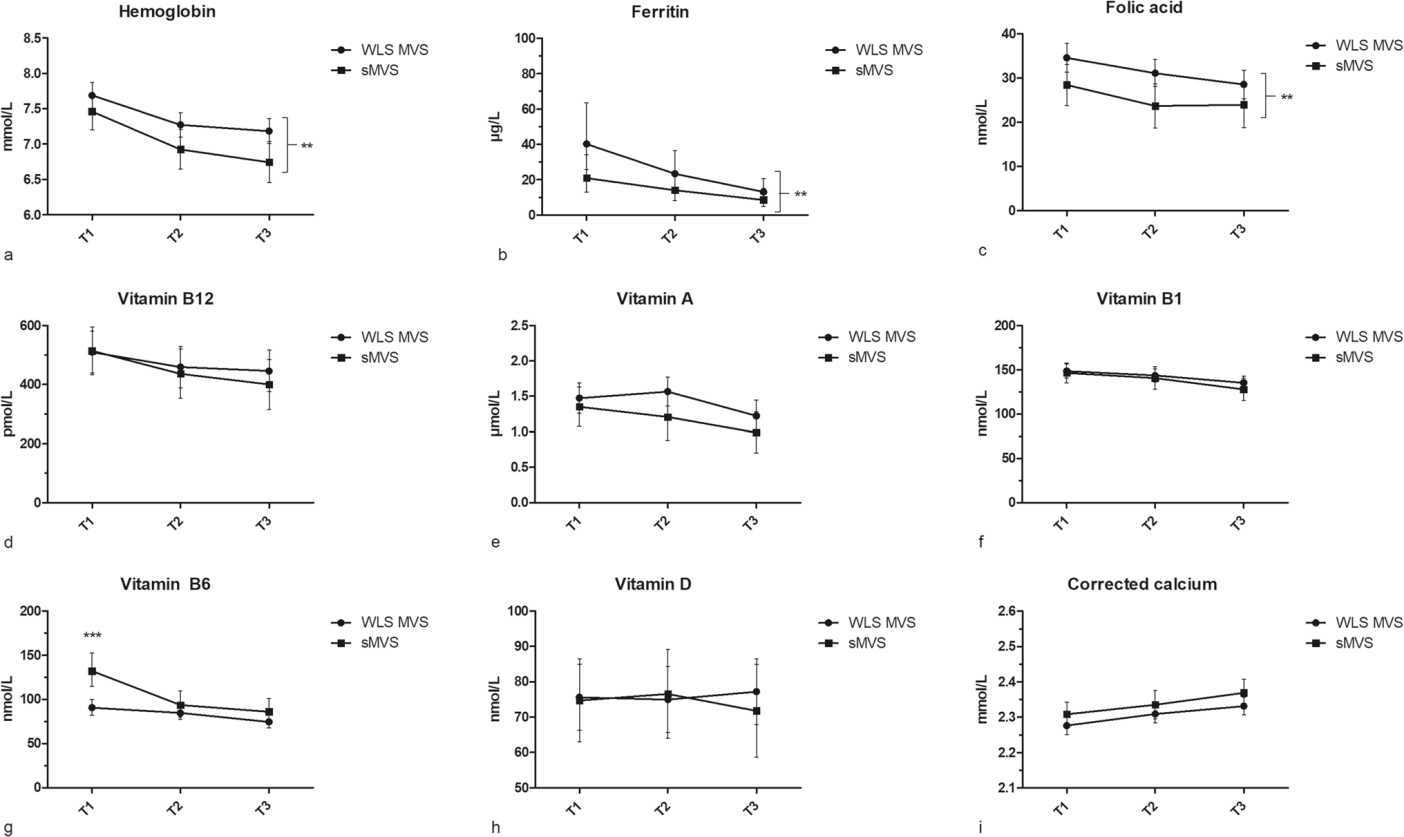
Serum concentrations for WLS-MVS users and sMVS users in the RYGB group across the trimesters of pregnancy (T1, T2, T3). Lines depict estimated marginal means and confidence intervals (error bars). **a** Hemoglobin. **Significantly higher serum levels for WLS-MVS compared to sMVS (*p*=0.01). **b** Ferritin. **Significantly higher serum levels for WLS-MVS compared to sMVS (*p*=0.003). **c** Folic acid. **Significantly higher serum levels for WLS-MVS compared to sMVS (*p*=0.01). **d** Vitamin B12. **e** Vitamin A. **f** Vitamin B1. **g** Vitamin B6. ***Significantly higher serum levels for sMVS compared to WLS-MVS at T1 (*p*<0.001). **h** Vitamin D. **i** Corrected calcium

During pregnancy, more participants used WLS-MVS compared to sMVS (T1: 69.6% vs. 30.4%, T2: 75.0% vs. 25.0%, T3: 75.3% vs. 24.7%). Overall, WLS-MVS users had significantly higher serum levels of hemoglobin, ferritin, and folic acid during pregnancy than sMVS users (*p* < 0.05 for all, Fig. 1a–c). This resulted in less iron deficiencies in the WLS-MVS group compared to the sMVS group during the second (29.6% vs. 55.6%, *p* = 0.047) and third trimester (36.5% vs. 72.2%, *p* = 0.01, Table 2). Similarly, anemia tended to be less prevalent in the WLS-MVS group (T1–T3: 11–13% vs. 17–33%, *p* > 0.05). The prevalence of low serum folic acid levels during pregnancy was comparable between the groups (2–12% vs. 0–6%). There was also a trend towards higher serum vitamin A concentrations in WLS-MVS users compared to sMVS users (1.42 µmol/L, 95% CI [1.27, 1.57] vs. 1.18 µmol/L, 95% CI [0.98, 1.39], *p* = 0.06). Similarly, the prevalence of low serum vitamin A levels tended to be lower in the WLS-MVS group (T1–T3: 14–22% vs. 25–46%, *p* > 0.05). Only one participant presented with an elevated serum vitamin A level during pregnancy (WLS-MVS, T2: 3.71 µmol/L). For vitamin B_6_, there was a significant interaction between MVS and trimester (*p* = 0.02, Fig. 1g). Compared to WLS-MVS users, sMVS users had higher serum vitamin B_6_ concentrations in the first trimester (90.6 nmol/L, 95% CI [82.0, 99.8] vs. 132.1 nmol/L, 95% CI [114.6, 152.4], *p* < 0.001), but levels decreased to similar concentrations in the second and third trimester. Accordingly, the prevalence of elevated serum vitamin B6 levels was significantly lower in the WLS-MVS group compared to the sMVS group during the first and second trimester, but not during the third trimester (T1: 32.6% vs. 61.9%, *p* = 0.02; T2: 13.0% vs. 43.8%, *p* = 0.01; T3: 12.5% vs. 22.2%, *p* = 0.44).

**Table 2:** Prevalence of serum levels below the lower reference limit during each trimester of pregnancy (T1, T2, T3) for WLS-MVS users versus sMVS users, stratified by type of MBS

Serum variables	Trimester	RYGB (*n* = 80)					SG (*n* = 39)				
		*n*	WLS MVS	*n*	sMVS	*p* value	*n*	WLS MVS	*n*	sMVS	*p* value
Hemoglobin	T1	*48*	6 (12.5)	*21*	7 (33.3)	0.05	*15*	0 (0.0)	*14*	1 (7.1)	0.48
	T2	*57*	7 (12.3)	*19*	4 (21.1)	0.45	*17*	1 (5.9)	*20*	1 (5.0)	0.99
	T3	*55*	6 (10.9)	*18*	3 (16.7)	0.68	*16*	1 (6.3)	*18*	2 (11.1)	0.99
Ferritin	T1	*47*	9 (19.1)	*21*	6 (28.6)	0.53	*14*	0 (0.0)	*11*	1 (9.1)	0.44
	T2	*54*	16 (29.6)	*18*	10 (55.6)	**0.047**	*17*	6 (35.3)	*19*	4 (21.1)	0.46
	T3	*52*	19 (36.5)	*18*	13 (72.2)	**0.01**	*16*	10 (62.5)	*18*	10 (55.6)	0.68
Folic acid	T1	*48*	1 (2.1)	*21*	1 (4.8)	0.52	*14*	0 (0.0)	*11*	0 (0.0)	–
	T2	*54*	3 (5.6)	*16*	0 (0.0)	0.99	*17*	2 (11.8)	*19*	1 (5.3)	0.59
	T3	*51*	6 (11.8)	*18*	1 (5.6)	0.67	*15*	2 (13.3)	*18*	4 (22.2)	0.67
Vitamin B_12_	T1	*48*	6 (12.5)	*21*	4 (19.0)	0.48	*14*	1 (7.1)	*13*	3 (23.1)	0.33
	T2	*54*	12 (22.2)	*16*	6 (37.5)	0.33	*17*	3 (17.6)	*19*	2 (10.5)	0.65
	T3	*52*	12 (23.1)	*17*	8 (47.1)	0.07	*15*	3 (20.0)	*18*	4 (22.2)	0.99
Vitamin A	T1	*20*	3 (15.0)	*12*	5 (41.7)	0.12	*8*	3 (37.5)	*8*	1 (12.5)	0.57
	T2	*22*	3 (13.6)	*8*	2 (25.0)	0.59	*14*	3 (21.4)	*13*	3 (23.1)	0.99
	T3	*18*	4 (22.2)	*11*	5 (45.5)	0.24	*10*	4 (40.0)	*14*	2 (14.3)	0.19
Vitamin B_1_	T1	*46*	1 (2.2)	*21*	0 (0.0)	0.99	*12*	0 (0.0)	*12*	0 (0.0)	–
	T2	*53*	0 (0.0)	*16*	0 (0.0)	–	*17*	1 (5.9)	*18*	0 (0.0)	0.49
	T3	*48*	0 (0.0)	*18*	1 (5.6)	0.27	*15*	2 (13.3)	*18*	0 (0.0)	0.20
Vitamin B_6_	T1	*46*	0 (0.0)	*21*	0 (0.0)	–	*12*	0 (0.0)	*12*	0 (0.0)	–
	T2	*54*	0 (0.0)	*16*	0 (0.0)	–	*17*	0 (0.0)	*18*	0 (0.0)	–
	T3	*48*	1 (2.1)	*18*	1 (5.6)	0.47	*15*	2 (13.3)	*18*	0 (0.0)	0.20
Vitamin D	T1	*48*	12 (25.0)	*21*	9 (42.9)	0.14	*15*	2 (13.3)	*13*	5 (38.5)	0.20
	T2	*54*	12 (22.2)	*17*	5 (29.4)	0.53	*17*	3 (17.6)	*19*	7 (36.8)	0.27
	T3	*51*	12 (23.5)	*18*	6 (33.3)	0.53	*16*	2 (12.5)	*18*	7 (38.9)	0.13
Calcium^a^	T1	*45*	3 (6.7)	*21*	0 (0.0)	0.55	*14*	0 (0.0)	*11*	0 (0.0)	–
	T2	*55*	2 (3.6)	*16*	0 (0.0)	0.99	*17*	0 (0.0)	*19*	0 (0.0)	–
	T3	*50*	0 (0.0)	*17*	1 (5.9)	0.25	*16*	0 (0.0)	*18*	0 (0.0)	–

Data are presented as frequency (percentage)

*RYGB*, Roux-en-Y gastric bypass; *SG*, sleeve gastrectomy; *WLS-MVS*, “weight loss surgery” multivitamin supplement; *sMVS*, standard multivitamin supplement (regular or prenatal supplements)

^a^Corrected for albumin levels

We did not find any differences in vitamin B_1_, vitamin B_12_, vitamin D, and calcium status between the two supplement groups.

### Nutrient Status and Supplement use after SG

Throughout pregnancy following SG, low maternal serum concentrations were frequently observed for ferritin (56.4%), folic acid (20.5%), vitamin B_12_ (35.9%), vitamin A (30.8%), and vitamin D (43.6%) and to a lesser extent for hemoglobin (12.8%). Low serum levels of calcium (0%) and vitamins B_1_ and B_6_ (5.1%) were rare.

**Fig. 2:**
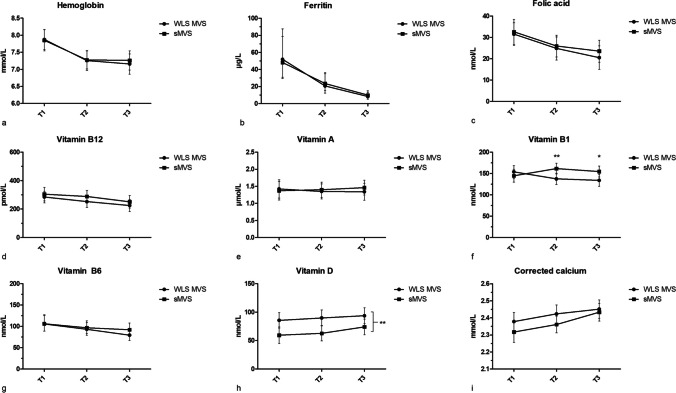
Serum concentrations for WLS-MVS users and sMVS users in the SG group across the trimesters of pregnancy (T1, T2, T3). Lines depict estimated marginal means and confidence intervals (error bars). **a** Hemoglobin. **b** Ferritin. **c** Folic acid. **d** Vitamin B12. **e** Vitamin A. **f** Vitamin B1. **Significantly higher serum levels for sMVS compared to WLS-MVS at T2 (*p*=0.01). *Significantly higher serum levels for sMVS compared to WLS-MVS at T3 (*p*=0.03). **g** Vitamin B6. **h** Vitamin D. **Significantly higher serum levels for WLS-MVS compared to sMVS (*p*=0.001). **i** Corrected calcium

During pregnancy, the number of participants using WLS-MVS was comparable to those using sMVS (T1: 51.7% vs. 48.3%, T2: 45.9% vs. 54.1%, T3: 50.0% vs. 50.0%). Overall, WLS-MVS users had significantly higher serum levels of vitamin D during pregnancy compared to sMVS users (89.7 nmol/L, 95% CI [77.6, 101.8] vs. 65.4 nmol/L, 95% CI [53.3, 77.4], *p* = 0.001, Fig. 2h). Similarly, low serum vitamin D levels tended to be less prevalent in the WLS-MVS group, although not statistically significant (T1–T3: 13–18% vs. 37–39%, *p* > 0.05, Table 2). For vitamin B_1_, there was a significant interaction between MVS and trimester (*p* = 0.02, Fig. 2f). Serum vitamin B_1_ concentrations were comparable in the first trimester, but slightly decreased over pregnancy in the WLS-MVS group, resulting in lower serum vitamin B_1_ levels in the second and third trimester compared to the sMVS group (T2: 137.4 nmol/L, 95% CI [124.2, 150.6] vs. 161.6 nmol/L, 95% CI [149.0, 174.1], *p* = 0.01; T3: 133.9 nmol/L, 95% CI [120.1, 147.7] vs. 154.7 nmol/L, 95% CI [141.9, 167.5], *p* = 0.03).

We did not find any differences in hemoglobin, ferritin, folic acid, vitamin B_12_, vitamin A, vitamin B_6_, and calcium status between the two supplement groups. There were no participants with an elevated serum vitamin A level during pregnancy after SG.

## Discussion

The aim of this observational cohort study was to explore differences in nutrient status among women using WLS-MVS versus sMVS during pregnancy following RYGB or SG. During pregnancy after RYGB, WLS-MVS users had higher serum levels of hemoglobin, ferritin, and folic acid and lower serum levels of vitamin B6 compared to sMVS users. Iron deficiencies as well as elevated serum vitamin B6 levels were also less prevalent in the WLS-MVS group. During pregnancy after SG, WLS-MVS users had higher serum levels of vitamin D, but lower serum levels of vitamin B_1_ compared to sMVS users.

To date, only one other (retrospective) study analyzing supplement use among 197 singleton pregnancies after RYGB has been performed, also showing higher serum levels of hemoglobin and ferritin for WLS-MVS users compared to users of prenatal supplements [19]. They additionally found higher serum vitamin D levels among WLS-MVS users. Despite the similar doses of vitamin D within the MVS used in both studies, differences in the use of additional calcium/vitamin D_3_ supplementation, season of sampling, and individual differences in supplement adherence and sun exposure could have impacted these findings. To the best of our knowledge, there are no other studies available that report on differences in nutrient status and the efficacy of WLS-MVS during pregnancy after MBS.

Overall, differences between the supplement groups were more pronounced within the RYGB group. Several factors could be involved including the small sample of pregnant women after SG and the higher non-compliance to supplement protocols within this group [20, 21]. Furthermore, pregnancy complications such as hyperemesis gravidarum only occurred in three women who all underwent SG and used WLS-MVS. Persistent vomiting can increase the risk of depleted serum concentrations, and therefore affect our findings. Nonetheless, future research is required as nutritional needs during pregnancy after SG may be different compared to the general SG population.

Our findings are in line with those observed in the general, non-pregnant bariatric patient population. Homan et al. also found higher serum levels of hemoglobin, ferritin, and folic acid and less anemia and iron deficiencies in WLS-MVS users compared to sMVS users 3 years after RYGB [14]. We additionally observed lower serum vitamin B_6_ levels in the WLS-MVS group, which may be explained by the slightly lower dose of vitamin B_6_ in the WLS-MVS used in the present study (0.6–0.98 mg (43–70%) RDA vs. 0.98 mg (70%) RDA). Nevertheless, serum vitamin B_6_ concentrations were near the upper reference limit in both groups. Although exposure to extremely high doses of vitamin B_6_ (> 50 mg/day) did not appear to be associated with an increased risk of major malformations during pregnancy [22], attention on elevated levels is needed as they may cause maternal peripheral neuropathy [23].

Two observational studies comparing nutrient status between WLS-MVS users and sMVS users in the general SG population also found higher serum vitamin D concentrations in WLS-MVS users [13, 15]. Remarkably, they also found higher serum vitamin B_1_ levels in the WLS-MVS group compared to the sMVS group, whereas we found the opposite during pregnancy in the present study [13, 15]. This may be explained by the prevalence of hyperemesis gravidarum within this subgroup. Persistent vomiting is a risk factor for thiamine deficiency, which can ultimately result in Wernicke’s encephalopathy [24, 25]. Still, serum vitamin B_1_ concentrations were far above the lower reference limit in both groups and low serum levels during pregnancy were rare (< 5%).

Consensus on recommended doses for supplementation during pregnancy after MBS has not yet been reached for most micronutrients, evidenced by the lack of evidence-based guidelines as well as the limited consistency across current recommendations [6]. This is concerning as the risk of micronutrient depletion posed by the MBS procedure may be even higher during pregnancy due to the associated physiologic changes. The present study confirmed that low maternal serum concentrations of hemoglobin, ferritin, folic acid, vitamin B_12_, vitamin D, and vitamin A are prevalent during pregnancy following MBS. Iron deficiency was observed in more than half of the women (RYGB: 60%, SG: 56%), indicating the need for additional iron supplementation in this population. However, oral iron supplements are often poorly tolerated [26]. Alternate day dosing of iron could provide an alternative solution as it significantly increases iron absorption and results in a lower incidence of gastrointestinal side effects compared with dosing iron every day [27, 28]. Intravenous iron administration should be considered in pregnant women with iron deficiency anemia who do not respond to or cannot tolerate oral iron supplementation during the second or third trimester [29]. For folic acid, low serum levels during pregnancy were slightly more prevalent after SG compared to RYGB (21% vs. 13%), but mean serum concentrations remained above the lower limit during pregnancy in all groups. It remains uncertain if additional supplementation for folic acid is required when high-dosed WLS-MVS are used and recommendations in clinical practice are inconsistent. Therefore, a critical review of folic acid requirement in pregnancy post-MBS is needed. Until then, the total supplementation dose should not exceed 1 mg per day if there are no specific medical needs for a high dose in order to prevent potential negative adverse effects from over-supplementation, such as masking of vitamin B_12_ deficiency [30, 31].

Next to the risks caused by low maternal concentrations of micronutrients, elevated serum levels can also have detrimental consequences for both mother and child. For vitamin A, supplementation with beta-carotene is preferred over the use of retinol during pregnancy due to the well-documented risk of teratogenic malformations [32]. We observed one case of hypervitaminosis A when using WLS-MVS for RYGB containing 800 µg retinol (13 weeks: 3.71 µmol/L). As information on dietary intake was unknown, it is difficult to ascertain whether this elevated level was caused by supplement intake and/or dietary intake. Overall, most WLS-MVS contain about 600–800 µg retinol, which is far below the safe upper level of 3000 µg as indicated by the European Food Safety Authority [33]. Besides, serum vitamin A concentrations markedly decreased within the lower range over the course of pregnancy and low serum vitamin A levels were prevalent in our study population (RYGB: 21%, SG: 31%). Previous research even reports up to 90% of vitamin A deficiencies after MBS [10]. Vitamin A deficiency has been shown to cause night blindness and is associated with fetal growth restriction [6, 7]. Therefore, continuing the use of WLS-MVS during pregnancy after MBS is considered safe and may even be preferred over the use of supplements containing beta-carotene because of its low conversion efficiency [34].

Nevertheless, we do acknowledge that this finding should be regarded with precaution and that regular monitoring of nutrient status during pregnancy is essential to detect any abnormal blood levels (both low and high) at an early stage.

The main strength of the present study is the availability of prospective data on MVS use across the three trimesters of pregnancy, including detailed information on supplement composition. Furthermore, as composition of WLS-MVS may differ per type of MBS procedure, we provided results for RYGB and SG separately.

However, our findings must also be interpreted in light of certain limitations. Most importantly, MVS use differed greatly within and between participants. Because of the relatively small study sample, we were limited to categorizing all MVS as either WLS-MVS or sMVS. Particularly for sMVS, differences in the type of MVS (prenatal vs. regular), composition, and dosing may have impacted the daily administered dose of nutrients. Greater sample sizes are required in order to obtain sufficient statistical power to address these variations. Moreover, underlying motivation or preferences regarding the use of MVS were not addressed in the present study.

We only used pregnancy-specific cut-off values for hemoglobin as uniform, evidence-based pregnancy-specific cut-offs for other micronutrients are lacking [35]. Due to the physiological decrease in serum levels caused by hemodilution and increasing demands of the growing fetus, the number of deficiencies in the present study may have been overestimated [12]. Although some guidelines on laboratory values in healthy pregnant women are available [36, 37], differences in used assays and population groups may limit their transferability to other centers and populations. Ideally, laboratories should provide locally validated reference ranges for pregnant women to recognize normal changes in laboratory values induced by pregnancy. Measuring direct or functional biomarkers (e.g., holotranscobalamin or methylmalonic acid for vitamin B_12_) could also increase our understanding with respect to functional deficiencies as the used assays in the present study might not have been sensitive enough to pick up deficiencies at lower levels, therefore, possibly underestimating its true prevalence.

Another limitation of the present study is the lack of comprehensive information on the preconception period, as women were only enrolled once they were pregnant. Furthermore, other factors including compliance with supplement protocols, dietary intake, and presence of severe complaints or complications (e.g., abdominal pain, internal herniation) during pregnancy may have also impacted maternal nutrient status during pregnancy and should be taken into account in future research.

Last, it should be noted that all study participants received secondary or tertiary obstetrician-led care which may limit the generalizability of our study results to women receiving primary midwife-led care.

To conclude, our study confirmed that low maternal concentrations of micronutrients are highly prevalent during pregnancy after MBS. This leads to greater challenges to reach nutritional requirements in these pregnancies, making optimal supplementation essential. Overall, we found a general trend towards higher serum levels over the course of pregnancy for women using specialized WLS-MVS compared to those using standard, prenatal supplementation. However, both low and elevated serum levels were still observed in both groups, emphasizing the need for regular assessment and monitoring of nutrient status at each trimester to detect abnormal levels at an early stage.

Future research is needed to investigate how supplementation strategies can be optimized individually for this high-risk population. Ideally, these studies should start before pregnancy, employ pregnancy-specific cut-off values, include direct or functional biomarkers of nutrient status, and take contributing factors as underlying motivation and preferences regarding multivitamin supplementation, compliance with supplement protocols, dietary intake, and complications during pregnancy into account.

Supplementary Information.

## Supplementary Information

Below is the link to the electronic supplementary material.

Supplementary file1 (DOCX 234 KB)

## Data Availability

The data that support the findings of this study are available from the corresponding author upon reasonable request.
