# Supplementary material for: Nutrient Status and Supplement Use During Pregnancy Following Metabolic Bariatric Surgery: A Multicenter Observational Cohort Study
Source: Obes Surg. 2024 Aug 14;34(10):3608–18. doi: 10.1007/s11695-024-07446-4 (PMC11464611; doi:10.1007/s11695-024-07446-4)
Supplement: Supplementary file 1 — Supplementary file1 (DOCX 234 KB) [file 11695_2024_7446_MOESM1_ESM.docx]

**Table S1.** Composition of the most frequently used WLS-MVS and sMVS.

|  | **WLS-MVS *SG*** | | **WLS-MVS *RYGB*** | | **sMVS *Prenatal*** | | **sMVS *Regular A*** | | **sMVS *Regular B*** | |
| --- | --- | --- | --- | --- | --- | --- | --- | --- | --- | --- |
|  | *Dose* | *RDA* | *Dose* | *RDA* | *Dose* | *RDA* | *Dose* | *RDA* | *Dose* | *RDA* |
| **Vitamin A** | 800 µg | 100% | 600 µg | 75% | 1200 µg^a^ | 0% | 800 µg | 100% | 533.4 µg | 66% |
| **Vitamin B1** | 2.75 mg | 250% | 2.75 mg | 250% | 1.1 mg | 100% | 1.1 mg | 100% | 1.1 mg | 100% |
| **Vitamin B2** | 1.7 mg | 121% | 2 mg | 143% | 1.4 mg | 100% | 1.4 mg | 100% | 1.4 mg | 100% |
| **Vitamin B3** | 25 mg | 156% | 32 mg | 200% | 16 mg | 100% | 16 mg | 100% | 16 mg | 100% |
| **Vitamin B5** | 9 mg | 150% | 18 mg | 300% | 6 mg | 100% | 6 mg | 100% | 6 mg | 100% |
| **Vitamin B6** | 1.5 mg | 107% | 0.98 mg | 70% | 1.4 mg | 100% | 1.4 mg | 100% | 1.4 mg | 100% |
| **Vitamin B8** | 150 µg | 300% | 100 µg | 200% | 50 µg | 100% | 50 µg | 100% | 50 µg | 100% |
| **Vitamin B11** | 500 µg | 250% | 600 µg | 300% | 400 µg | 200% | 200 µg | 100% | 200 µg | 100% |
| **Vitamin B12** | 100 µg | 4000% | 350 µg | 14000% | 2.5 µg | 100% | 2.5 µg | 100% | 2.5 µg | 100% |
| **Vitamin C** | 100 mg | 125% | 120 mg | 150% | 40 mg | 50% | 80 mg | 100% | 80 mg | 100% |
| **Vitamin D3** | 75 µg | 1500% | 75 µg | 1500% | 10 µg | 200% | 5 µg | 100% | 3.4 µg | 66% |
| **Vitamin E** | 15 mg | 125% | 24 mg | 200% | 6 mg | 50% | 12 mg | 100% | 12 mg | 100% |
| **Vitamin K1** | - | - | - | - | - | - | 75 µg | 100% | - | - |
| **Calcium** | - | - | - | - | 120 mg | 15% | 160 mg | 20% | 80 mg | 10% |
| **Chrome** | 40 µg | 100% | 160 µg | 400% | 20 µg | 50% | 25 µg | 63% | 40 µg | 100% |
| **Iodine** | 150 µg | 100% | 150 µg | 100% | 150 µg | 100% | 150 µg | 100% | 100 µg | 66% |
| **Copper** | 1.9 mg | 190% | 3 mg | 300% | 1 mg | 100% | 1.5 mg | 150% | 1 mg | 100% |
| **Magnesium** | - | - | - | - | 56.25 mg | 15% | 125 mg | 33% | 37.6 mg | 10% |
| **Manganese** | 3 mg | 150% | 3 mg | 150% | 1 mg | 50% | 1 mg | 50% | 2 mg | 100% |
| **Molybdenum** | 50 µg | 100% | 112.4 µg | 225% | 25 µg | 50% | 25 µg | 50% | 50 µg | 100% |
| **Selenium** | 55 µg | 100% | 105 µg | 191% | 55 µg | 100% | 25 µg | 45% | 55 µg | 100% |
| **Iron** | 28 mg | 200% | 70 mg | 500% | 16.1 mg | 115% | 14 mg | 100% | 14 mg | 100% |
| **Zinc** | 28 mg | 280% | 22.5 mg | 225% | 10 mg | 100% | 15 mg | 150% | 6.6 mg | 66% |

*WLS-MVS SG,* ‘weight loss surgery’ multivitamin supplement for sleeve gastrectomy; *WLS-MVS RYGB,* ‘weight loss surgery’ multivitamin supplement for Roux-en-Y gastric bypass; *sMVS*, standard multivitamin supplement; *RDA,* recommended daily allowance.

^a^ Beta-carotene.

**Table S2.** Reference values of the evaluated micronutrients for each hospital.

| **Serum variables** | **Reference values** | | |
| --- | --- | --- | --- |
|  | **RHA** | **MMC** | **HGV** |
| **Hemoglobin**^a^ | T1: 7.1 mmol/L  T2: 6.5 mmol/L  T3: 6.3 mmol/L | T1: 7.1 mmol/L  T2: 6.5 mmol/L  T3: 6.3 mmol/L | T1: 7.1 mmol/L  T2: 6.5 mmol/L  T3: 6.3 mmol/L |
| **Ferritin** | 10-291 µg/L | 13-150 µg/L | 13-150 µg/L |
| **Folic acid** | > 12.2 nmol/L^b^ | > 8 nmol/L | 7-40 nmol/L |
| **Vitamin B12**^c^ | >200 pmol/L | >200 pmol/L | >200 pmol/L |
| **Vitamin A** | 1.05-2.80 µmol/L | *NA* | 1.13-2.72 µmol/L |
| **Vitamin B1** | 95-175 nmol/L | 66.5-200 nmol/L | 90-200 nmol/L |
| **Vitamin B6** | 25-100 nmol/L | 35-110 nmol/L | 51-183 nmol/L |
| **Vitamin D** | >50 nmol/L | >50 nmol/L | >50 nmol/L |
| **Calcium**^d^ | 2.23-2.55 mmol/L | 2.15-2.55 mmol/L | 2.23-2.55 mmol/L |

^a^ Reference value during pregnancy according to The Royal Dutch Organisation of Midwives (2010).

^b^ Reference value before 8-1-2019 was 5-35 nmol/l.

^c^ Reference value after bariatric surgery.

^d^ Corrected for albumin levels.

**Table S3.** Serum concentrations during each trimester of pregnancy (T1, T2, T3) for WLS users versus sMVS users, stratified by type of MBS.

| **Serum variables** | **Trimester** | **RYGB (n=80)** | | | | **SG (n=39)** | | | |
| --- | --- | --- | --- | --- | --- | --- | --- | --- | --- |
|  |  | *n* | **WLS-MVS** | *n* | **sMVS** | *n* | **WLS-MVS** | *n* | **sMVS** |
| **Hemoglobin**  (mmol/L) | T1 | *48* | 7.7 ± 0.7 | *21* | 7.4 ± 1.0 | *15* | 7.9 ± 0.5 | *14* | 7.9 ± 0.5 |
|  | T2 | *57* | 7.3 ± 0.7 | *19* | 7.0 ± 0.8 | *17* | 7.2 ± 0.6 | *20* | 7.3 ± 0.5 |
|  | T3 | *55* | 7.2 ± 0.6 | *18* | 6.8 ± 0.8 | *16* | 7.1 ± 0.8 | *18* | 7.3 ± 0.6 |
| **Ferritin**  (µg/L) | T1 | *47* | 40.0 (12.0-90.0) | *21* | 20.0 (7.5-59.0) | *14* | 56.0 (27.8-99.5) | *11* | 38.0 (20.0-77.0) |
|  | T2 | *54* | 20.0 (11.0-48.5) | *18* | 12.0 (8.0-30.3 | *17* | 20.0 (6.5-45.5) | *19* | 23.0 (11.0-45.0) |
|  | T3 | *52* | 16.5 (9.3-32.0) | *18* | 8.5 (7.0-14.3) | *16* | 9.0 (6.3-13.5) | *18* | 9.0 (7.0-15.0) |
| **Folic acid**  (nmol/L) | T1 | *48* | 34.2 ± 11.6 | *21* | 27.3 ± 12.2 | *14* | 33.0 ± 9.8 | *11* | 31.4 ± 11.6 |
|  | T2 | *54* | 30.0 ± 11.4 | *16* | 29.7 ± 10.1 | *17* | 25.8 ± 13.3 | *19* | 25.9 ± 12.6 |
|  | T3 | *51* | 28.1 ± 13.6 | *18* | 28.0 ± 12.5 | *15* | 21.9 ± 11.8 | *18* | 23.0 ± 13.5 |
| **Vitamin B12**  (pmol/L) | T1 | *48* | 320.0 (254.5-436.8) | *21* | 320.0 (235.0-558.5) | *14* | 284.0 (220.3-356.3) | *13* | 303.0 (211.0-531.5) |
|  | T2 | *54* | 280.0 (207.5-380.0) | *16* | 233.0 (167.8-587.8) | *17* | 250.0 (229.0-295.0) | *19* | 283.0 (223.0-344.0) |
|  | T3 | *52* | 267.5 (202.5-379.0) | *17* | 200.0 (172.0-447.5) | *15* | 237.0 (200.0-267.0) | *18* | 282.5 (212.5-339.5) |
| **Vitamin A**  (µmol/L) | T1 | *20* | 1.49 ± 0.43 | *12* | 1.34 ± 0.47 | *8* | 1.31 ± 0.38 | *8* | 1.45 ± 0.29 |
|  | T2 | *22* | 1.58 ± 0.69 | *8* | 1.24 ± 0.39 | *14* | 1.33 ± 0.36 | *13* | 1.41 ± 0.43 |
|  | T3 | *18* | 1.22 ± 0.31 | *11* | 1.03 ± 0.38 | *10* | 1.37 ± 0.45 | *14* | 1.47 ± 0.48 |
| **Vitamin B1**  (nmol/L) | T1 | *46* | 149.9 ± 28.3 | *21* | 143.6 ± 24.2 | *12* | 159.9 ± 21.4 | *12* | 143.4 ± 24.3 |
|  | T2 | *53* | 143.6 ± 29.5 | *16* | 143.3 ± 27.2 | *17* | 138.1 ± 26.8 | *18* | 160.2 ± 34.0 |
|  | T3 | *48* | 137.0 ± 26.9 | *18* | 131.3 ± 29.1 | *15* | 133.9 ± 31.8 | *18* | 153.7 ± 26.7 |
| **Vitamin B6**  (nmol/L) | T1 | *46* | 91.5 (73.0-113.0) | *21* | 111.0 (90.5-182.5) | *12* | 105.0 (93.8-130.5) | *12* | 104.0 (76.5-135.3) |
|  | T2 | *54* | 81.5 (74.8-95.3) | *16* | 97.5 (76.5-122.0) | *17* | 96.0 (66.5-125.5) | *18* | 100.5 (70.3-108.3) |
|  | T3 | *48* | 79.0 (57.5-92.8) | *18* | 85.5 (71.0-101.5) | *15* | 99.0 (77.0-105.0) | *18* | 80.0 (63.8-103.8) |
| **Vitamin D**  (nmol/L) | T1 | *48* | 80.0 ± 37.8 | *21* | 63.7 ± 33.2 | *15* | 88.1 ± 31.1 | *13* | 59.2 ± 30.8 |
|  | T2 | *54* | 80.1 ± 34.2 | *17* | 72.8 ± 33.8 | *17* | 87.9 ± 30.9 | *19* | 65.1 ± 32.9 |
|  | T3 | *51* | 82.7 ± 36.9 | *18* | 72.2 ± 34.1 | *16* | 90.9 ± 32.3 | *18* | 68.0 ± 37.4 |
| **Calcium**  (mmol/L) | T1 | *46* | 2.27 ± 0.10 | *21* | 2.31 ± 0.10 | *14* | 2.32 ± 0.08 | *11* | 2.31 ± 0.10 |
|  | T2 | *55* | 2.20 ± 0.09 | *16* | 2.21 ± 0.11 | *17* | 2.26 ± 0.08 | *19* | 2.23 ± 0.10 |
|  | T3 | *50* | 2.18 ± 0.08 | *17* | 2.18 ± 0.09 | *16* | 2.24 ± 0.10 | *18* | 2.25 ± 0.11 |

Data are presented as mean ± SD or median (Q1-Q3).

*RYGB*, Roux-en-Y gastric bypass; *SG*, sleeve gastrectomy; *WLS-MVS,* ‘weight loss surgery’ multivitamin supplement; *sMVS*, standard multivitamin supplement (regular or prenatal supplements).


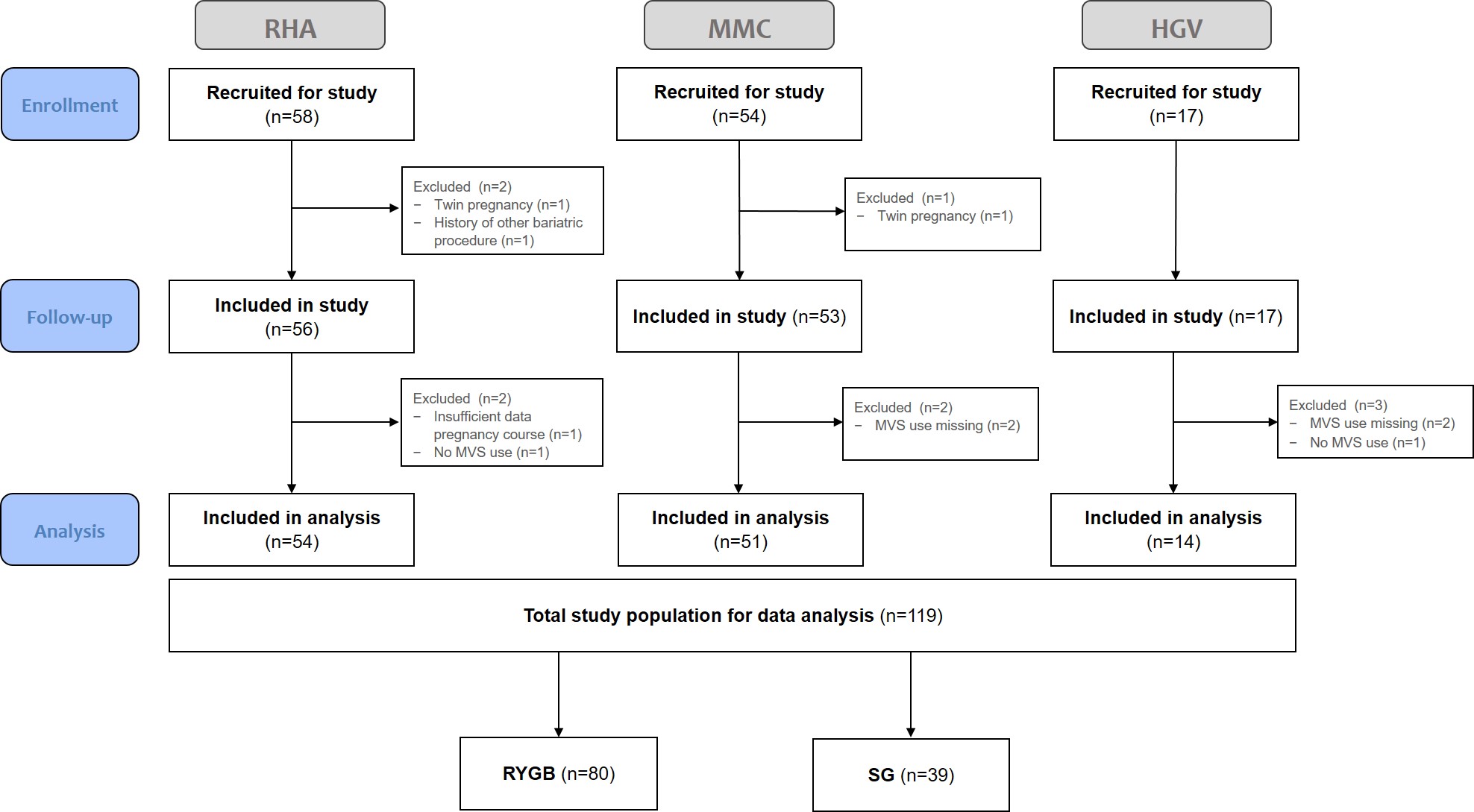


**Figure S1.** Flowchart inclusion
